# GPP (Composition of *Ganoderma lucidum* Poly-saccharides and *Polyporus umbellatus* Poly-saccharides) Enhances Innate Immune Function in Mice

**DOI:** 10.3390/nu11071480

**Published:** 2019-06-28

**Authors:** Qing Huang, Liyuan Li, Huiling Chen, Qingfei Liu, Zhao Wang

**Affiliations:** MOE Key Laboratory of Protein Science, School of Pharmaceutical Sciences, Tsinghua University, Beijing 100084, China

**Keywords:** *Ganoderma lucidum* polysaccharides, *Polyporus umbellatus* polysaccharides, GPP, Immunoregulation

## Abstract

Modern research has found that *Ganoderma lucidum* polysaccharides (GLP) and *Polyporus umbellatus* polysaccharides (PUP) mainly exhibit immunoregulation. However, the immune function of a polysaccharide composition consisting of GLP and PUP has not been studied. In this study, we developed a polysaccharide composition consisting of GLP and PUP in a ratio of 3:1 (named GPP). The immunoregulation of GPP was detected in RAW264.7 macrophages. Then, the acute oral toxicity of GPP and the effect of GPP on immunoregulation in mice was detected. The results showed that GPP enhanced the function of macrophage RAW264.7 cells through improving phagocytic ability, nitric oxide (NO) production and the mRNA expression of inducible nitric oxide synthase (iNOS) and tumor necrosis factor (TNF)-α. GPP belonged to the non-toxic grade in mice. Moreover, GPP significantly improved macrophage phagocytic function and the activity of natural killer (NK) cells after being administered to mice at a dose of 0, 3.6, 120, 360 mg/kg body weight (mg/kg BW) orally for 30 days. Taken together, these findings suggested that GPP moderately regulated immune function in mice, which contributes to the further development and utilization of GLP and PUP in immune function.

## 1. Introduction

Polysaccharides, a class of natural macromolecule polymers which consist of more than 10 monosaccharides linked with glycosidic bonds, have a wide range of biological activities [1]. Edible mushroom polysaccharides (EMPs) are a member of the polysaccharide family. As natural compounds, EMPs are very good candidates for developing functional foods and nutraceuticals due to their various biological activities and relatively low toxicity [2]. EMPs have shown a wide range of biological functions, such as anti-tumor, anti-bacterial, antioxidant, antiviral, regulating gut microbiota and immunomodulatory activities [2,3,4]. Among them, immunomodulatory activity of polysaccharides is one of the hotspots in current research. A variety of EMPs are involved in the regulation of innate immunity and acquired immunity by influencing various important immune organs [2,5], immune cells and immune factors [3]. Some edible mushrooms are also involved in Chinese herbal medicine such as *Ganoderma lucidum* and *Polyporus umbellatus*. The mechanisms of Chinese herbal medicine polysaccharides are considered to participate in the activity regulation of T cells, B cells, macrophages, dendritic cells and natural killer (NK) cells, binding to specific receptors on the cell surface, activating various signal transduction pathways in the cell, stimulating cytokine or antibody secretion, and thus regulating the immune function of the body at multiple levels, multiple channels and multiple targets [6]. In recent years, there have been many studies focusing on the immunomodulation of polysaccharides from the same source, but few studies have focused on the functions of the immune system of polysaccharide composition from different sources. However, considering the complex structure of polysaccharides, the immunomodulation of polysaccharides from different sources may be different. Therefore, whether the composition of polysaccharides from different sources can coordinate immunomodulation has been of interest.

Both *G. lucidum* and *P. umbellatus* are valuable edible and medicinal mushrooms with a history of use for more than 2000 years in China in the treatment diseases and regulation the body balance [7,8,9]. Ancient Chinese medical scholars suggested that *G. lucidum* had the activity of “Fuzheng Guben”, which means it could strengthen body resistance and consolidate the constitution of patients [10], while *P. umbellatus* could induce diuresis and excrete dampness [11]. According to traditional Chinese medicine theory, the combination of *G. lucidum* and *P. umbellatus* was meaningful because the combination had the characteristics of supplement without causing stagnation. Current research has found that *G. lucidum* and *P. umbellatus* polysaccharides mainly exhibited immunoregulation, which adjusted the function of a variety of immune cells including T cells, B cells, macrophages, dendritic cells and NK cells [8,12,13]. *G. lucidum* polysaccharides (GLP) and *P. umbellatus* polysaccharides (PUP) in combination with chemotherapy drugs enhance efficacy and reduce toxic side effects via the immune system, respectively [14,15]. Above all, it was hypothesized that the combination of GLP and PUP may regulate the immune function more effectively.

Macrophages are immune cells derived from progenitor cells in the bone marrow [16] which play an important bridging role in innate immunity and acquired immunity [17]. As a key member of the innate immune defense front of the body, macrophages exist in almost all tissues, and their main function is to engulf and degrade dead cells, cell debris and foreign pathogens, and coordinate the inflammatory process [18,19]. In addition, macrophages are capable of presenting antigens, secreting a variety of active substances, regulating the local microenvironment and other physiological functions [20]. Currently, it has been reported that GLP and PUP obtained by different extraction methods affected the activity of macrophages. For example, a kind of GLP peptide promoted the polarization of the M1 macrophage induced by lipopolysaccharide (LPS) [21]. In addition, a kind of GLP with a molecular weight of 15.1 kDa induced the differentiation and nitric oxide (NO) production of macrophages [3]. A 95% pure PUP promoted NO production, tumor necrosis factor (TNF)-α and interleukin-1 (IL)-1 by activating the function of mouse peritoneal macrophages [9]. However, no studies have shown whether the two can more effectively regulate macrophage function.

In this paper, a compound consisting of water-soluble GLP and water-soluble PUP in a ratio of 3:1 named GPP was used to study its effects on immunoregulation. The results showed that GPP enhanced the function of macrophages by improving phagocytic function, promoting NO production, and increasing the RNA expression of inducible nitric oxide synthase (iNOS) and TNF-α. GPP was slightly better than GLP in enhancing the function of macrophages although without statistical significance. GPP significantly enhanced the function of macrophages compared with PUP in a certain concentration range. Moreover, GPP significantly improved macrophages’ phagocytic function and the activity of NK cells after being administered to mice orally for 30 days. Based on the above experimental results, it was suggested that GPP played some role in regulating innate immune function in mice. Our results contribute to further developing and utilizing GLP and PUP in immunomodulation.

## 2. Materials and Methods 

### 2.1. Materials

GLP, PUP and sheep red blood cells (SRBC) were purchased from Yuanye Biotechnology (Shanghai, China). Lipopolysaccharide (LPS), NO assay kits, Neutral Red Cell Proliferation, Cytotoxicology Assay Kits were obtained from Beyotime Biotechnology (Shanghai, China). Fetal bovine serum (FBS) was obtained from Invitrogen Corporation (Carlsbad, CA, USA). Dulbecco’s modified Eagle Medium (DMEM) and penicillin/streptomycin were purchased from Corning Incorporated (Corning, NY, USA). Eastep^®^ Super Total RNA Extraction Kit was purchased from Promega Co., Ltd, (Madison, Wisconsin, USA). Real-time quantitative PCR (qPCR)-related kits, FastQuant RT kit and Super Real PreMix Plus were purchased from Tiangen Biotech CO., Ltd. (Beijing, China). Roswell Park Memorial Institute (RPMI) 1640 was obtained from Gibco (Carlsbad, CA, USA). Concanavalin A was purchased from Beijing Solarbio Science & Technology Co., Ltd (Beijing, China). RAW264.7 cells were obtained from Dr. Hanqiu Zheng (Department of Basic Medical Sciences, Tsinghua University, China). YAC-1 tumor cells were obtained from Prof. Weidong Hao (School of Public Health, Peking University, China). All other chemicals were of analytical grade and used as received.

### 2.2. RAW264.7 Cells Culture and Sample Preparation

FBS was inactivated at 56 °C for 30 min before use. RAW264.7 Cells were cultured in DMEM medium supplemented with 10% inactivated FBS and 100 units/mL penicillin–streptomycin and kept at 37 °C in a humidified incubator containing 5% CO_2_. GPP comprised GLP and PUP in a ratio of 3:1. GLP, PUP and GPP were dissolved in medium which was used to culture RAW264.7 cells, and then filtered by 0.2 μm membrane filter. When confluence reached 80%, cells were washed by PBS twice and harvested by trypsin, and then centrifugated at 800 × g for 5 min to discard supernatant. Cells were re-suspended by DMEM complete medium at a density of 4 × 10^5^ cells mL^−1^ for the following experiments. 

### 2.3. Macrophage Phagocytosis Assay

Neutral red uptake assay was used to detect the efficacy of GPP on the phagocytosis of macrophages. Briefly, RWA264.7 cells were seeded into 96-well plates at the density of 4 × 10^4^ cells/well and incubated overnight. Then, cells were exposed to different concentrations (50, 100 and 200 μg/mL) of GLP, PUP, GLP respectively, or 5 μg/mL LPS for 24 h. Supernatant was discarded and each well was supplemented with 200 μL fresh DMEM complete medium and 20 μL neutral red solution for a further 2 h incubation. Cells were washed by PBS twice to remove neutral red, and then 200 μL cell lysis solution was added. The cells’ absorbance values at 560 nm were acquired after cracking the shaker at room temperature for 10 min. 

### 2.4. NO Assay

Griess assay was used to detect the effect of GPP on the function of NO release in macrophages. Briefly, the method of RWA264.7 cells culture and sample treatment were the same as the macrophage phagocytosis assay. Supernatant was replaced by 100 μL fresh DMEM complete medium for a further 2 h incubation after incubation with GLP, PUP, GPP or LPS for 24 h. Then, 50 μL Griess reagent I and 50 μL Griess reagent II were added into each well. The standard curve was made following the protocol at the same time. The absorbance value at 560 nm was then measured. The relative NO release in each group was calculated according to the standard curve. 

### 2.5. Real-time Quantitative PCR

RAW264.7 cells were seeded into six-well plates at a density of 1 × 10^6^ cells per well and incubated overnight. Then, cells were exposed to 100 μg/mL GLP, PUP, GPP respectively, or 5 μg/mL LPS for 24 h. Cells were washed by cold PBS twice and then harvested by gentle scraping. Then, they were centrifugated at 12000 × rpm for 1 min to obtain cells. For qPCR analysis, total RNA was extracted using at Eastep^®^ Super Total RNA Extraction Kit according to the manufacturer’s protocol. cDNA was obtained by a FastQuant RT Kit and real-time PCR analysis was conducted with SuperReal PreMix Plus according to the two-step reaction program. qPCR analysis was conducted using primers as follows: *Tnf* forward 5′-CAGGCGGTGCCTATGTCTC-3′, reverse 5′-CGATCACCCCGAAGTTCAGTAG-3′, *nitric oxide synthase 2* (*Nos2*) forward 5′-GGAGTGACGGCAAACATGACT-3′, reverse 5′-TCGATGCACAACTGGGTGAAC-3′, *IL-10* forward 5′-GCTCTTACTGACTGGCATGAG-3′, reverse 5′-CGCAGCTCTAGGAGCATGTG-3′, *IL-6* forward 5′-CTGCAAGAGACTTCCATCCAG-3′, reverse 5′-AGTGGTATAGACAGGTCTGTTGG-3′, *actin* forward 5′-GGCTGTATTCCCCTCCATCG-3′, reverse 5′-CCAGTTGGTAACAATGCCATGT -3′.

### 2.6. Animals and GPP Treatment

ICR mice weighing about 18–22 g were purchased from the laboratory animal center of Tsinghua University (Beijing, China) and were housed in cages with standard laboratory food and water *ad libitum*. The house was maintained under an ambient temperature of 22 ± 2 °C and a 12 h light/dark cycle. All animal experiments were conducted according to the relevant guidelines and regulations and with the approval of the Institutional Ethical Committee of China and of the Institutional Animal Care and Use Committee in Tsinghua University. The ethical approval code for the animal experiments was 18-WZ1. All mice had an adaptive phase of 5 days before starting the experiment. 

The acute oral toxicity experiment was in accordance with Chinese national food safety standards and acute oral toxicity guidelines. Briefly, animals were divided into a male group and a female group (N = 10) and orally treated with a maximal dosage of GPP (16 g/kg) for 14 days. General behavior and mortality were observed and recorded during the experiment. 

In the immune function detection experiment, female mice were randomly divided into four groups (N = 12). GPP was dissolved by pure water. The control groups were administered with pure water while the other three mice groups received 3.6, 12, and 360 mg/kg body weight (mg/kg BW) doses of GPP which were 3, 10 and 30 times the human recommended dosage, respectively. All groups were administered test substances at 0.1 mL/10 g BW per day by oral gavage for 30 days before the following experiments. The body weight gain was recorded during the experiments.

### 2.7. Measurement of Immune Organ Indexes 

After the last time of GPP treatment, mice were weighed and sacrificed. Then the spleen and thymus were immediately removed and weighed. The spleen or thymus index was calculated according to the following formula:

Thymus or spleen index = Thymus or spleen weight (mg)/Body weight (g)

### 2.8. Measurement of Phagocytotic Function of Peripheral Phagocytes in Mice

The method of detecting the phagocytotic capacity of peripheral phagocytes in mice was conducted according to the method as previously described [22]. Briefly, after the last GPP treatment, mice were immunized with 1 mL 2% (*v*/*v*, diluted with saline) chicken red blood cells (CRBC) by intraperitoneal injection. After 30 min, the mice were sacrificed. Two milliliters of saline were intraperitoneally injected immediately. Then, the mouse abdomens were gently pressed for 20 s. The 1 mL of lavage fluid containing peritoneal cells was collected with straws to evenly smear on two glass slides and incubated at 37 °C for 30 min in a wet environment. Then, the glass slides were washed with physiological saline to remove unattached cells and dried at room temperature. The cells on the slide was fixed with acetone methanol (1:1) solution and stained with 4 % Giemsa for 3 min, and then washed with water and dried at room temperature. The number of phagocytic cells ingesting CRBC and macrophages were observed and calculated under oil microscope. The percentage phagocytosis phagocytic index was calculated according to the following formula:

$$\mathrm{Percentage}\;\mathrm{phagocytosis}\;=\frac{\mathrm{number}\;\mathrm{of}\;\mathrm{phagocytic}\;\mathrm{cells}\;\mathrm{ingesting}\;\mathrm{CRBC}}{\mathrm{number}\;\mathrm{of}\;\mathrm{macrophages}}\;\times 100$$

$$\mathrm{Phagocytic}\;\mathrm{index}=\frac{\mathrm{Number}\;\mathrm{of}\;\mathrm{phagocytized}\;\mathrm{CRBC}}{\mathrm{number}\;\mathrm{of}\;\mathrm{macrophages}}$$

### 2.9. Measurement of NK Cell Activity in Mice

The method of detecting NK cell activity by lactate dehydrogenase (LDH) releasing assay was performed according to the method as previously described [23]. Briefly, the spleens were immediately removed and placed in Hanks medium. The suspension of splenocytes was obtained by gently grinding the mice spleen and passing of the spleens through a steel mesh. The erythrocytes were lysed with red cell lysis buffer, and splenocytes were collected by centrifugation at 1000 × g for 5 min. Splenocytes were resuspended by RPMI1640 medium supplemented with 10% inactivated FBS and 100 units/mL penicillin–streptomycin and kept at 37 °C in a humidified incubator containing 5% CO_2_. The concentration of splenocytes was adjusted to 5 × 10^6^ cells/mL. YAC-1 cells were prepared as target cells and the density was adjusted to 0.1 × 10^6^ cells/mL. 100 μL YAC-1 cells and 100 μL splenocytes (effector cell) were added to a 96-well plate (the ratio of effector cells to target cells was 50:1). At the same time, the target cell spontaneous release control (100 μL YAC-1 cells plus 100 μL media) and target cell maximum release control (100 μL YAC-1 cells plus 100 μL 1% NP40) were prepared. After being incubated at 37 °C in a humidified incubator containing 5% CO2 for 4 h, the 96-well plate was centrifuged at 1500 rpm for 5 min. Then, 100 μL supernatant was transferred into a new 96-well plate mixed with 100 μL LDH substrate for a 5 min reaction. Thirty microliters of l mol/L HCI was used to stop the reaction. Absorbance values at 490 nm were then measured. NK cell activity was calculated according to the following formula:

$$\mathrm{NK}\;\mathrm{cell}\;\mathrm{viability}\;(\%)\;=\frac{{\mathrm{OD}}_{\mathrm{sample}}\;-\;{\mathrm{OD}}_{\mathrm{YAC}-1\;\mathrm{cell}\;\mathrm{spon}\;\mathrm{aneous}\;\mathrm{release}}}{{\mathrm{OD}}_{\mathrm{YAC}-1\;\mathrm{cell}\;\mathrm{maximun}\;\mathrm{release}}}\;\times 100$$

### 2.10. Statistical Analysis

Results were expressed as mean ± SEM. For the in vitro experiment, the result reported was from at least three independent experiments (N = 6 for each group). For the in vivo animal experiment, each group contained 10 or 12 mice. Statistical analysis was performed using SPSS software (SPSS Inc., Chicago, IL, USA) through one-way ANOVA. *p* < 0.05 was considered to be a statistically significant difference. The histogram was made by GraphPad Prism 6.0 software (GraphPad Software, Inc., San Diego, CA, USA).

## 3. Results

### 3.1. GPP Increased Phagocytosis and NO Production in RAW264.7 Cells

**Figure 1:**
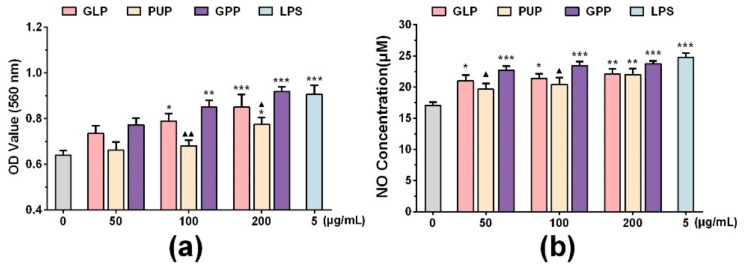
The effect of *Ganoderma lucidum* polysaccharides (GLP), *Polyporus umbellatus* polysaccharides (PUP) and a polysaccharide composition consisting of GLP on phagocytosis and NO production in RAW264.7 cells, respectively. (**a**) Phagocytosis of macrophages was detected by neutral red assay. (**b**) The release of nitric oxide (NO) was detected by Griess assay in macrophages. Data were expressed as mean ± SEM, * *p* < 0.05, ** *p* < 0.01, *** *p* < 0.001 vs. control group, ^▲^ *p* < 0.05, ^▲▲^ *p* < 0.01 vs. GPP group.

RAW264.7 cells were derived from BALB/c mice cells infected with Abelson leukemia virus which were considered to be a suitable model to study the function of macrophage because of their macrophage characteristics [3,24]. To compare the effects of GLP, PUP and GPP on the function of macrophages, their effects at different concentrations (50, 100, 200 μg/mL) on phagocytosis ability and NO production in RAW264.7 cells were detected by neutral red assay and Griess assay, respectively. The results showed that LPS as a positive control significantly enhanced the ability of phagocytosis and released NO (*p* < 0.001) in RAW264.7 cells (Figure 1). Compared with the control group, the experimental groups demonstrated enhanced ability of macrophage phagocytosis, and released NO in a dose-dependent manner. Compared with the GLP group, GPP had the tendency to increase the ability of the phagocytosis of neutral red and the release of NO. Compared with the PUP group, GPP significantly enhanced the ability of macrophages to phagocytose neutral red at the concentrations of 100 and 200 μg/mL (*p* < 0.01, *p* < 0.05), and significantly enhanced the ability of macrophages to release NO at the concentrations of 50 and 100 μg/mL (*p* < 0.05).The results showed that GPP was slightly better than GLP at enhancing the function of macrophages, although without statistical significance. Moreover, GPP significantly enhanced the function of macrophages compared with PUP in a certain concentration range.

### 3.2. GPP Increased the mRNA Expression of INOS, TNF-α and IL-10

**Figure 2:**
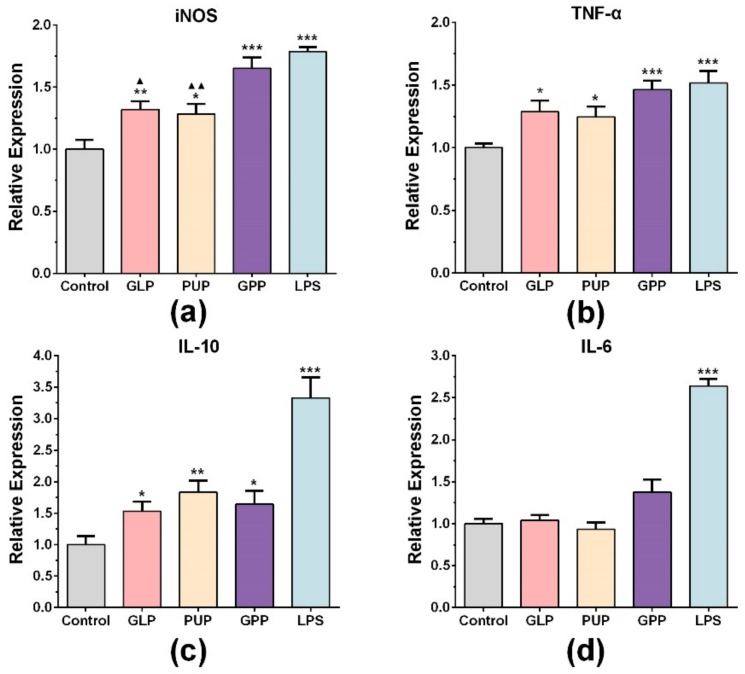
The effect of GLP, PUP and a polysaccharide composition consisting of GLP and PUP in a ratio of 3:1 (GPP) on iNOS, TNF-α, IL-10 and IL-6 mRNA expression, respectively. The mRNA expression level of iNOS (**a**), TNF-α (**b**), IL-10 (**c**) and IL-6 (**d**). Data were expressed as mean ± SEM, * *p* < 0.05, ** *p* < 0.01, *** *p* < 0.001 vs. control group, ^▲^ *p* < 0.05, ^▲▲^ *p* < 0.01 vs. GPP group.

To further explore the effect of GPP on NO release function, the mRNA expression of iNOS at the concentrations of 100 μg/mL of GLP, PUP and GPP were determined by RT-qPCR, respectively. As shown in Figure 2a, LPS significantly increased the mRNA expression of iNOS. Compared with the control group, the mRNA expression of iNOS in experimental groups was also significantly enhanced, and the mRNA expression of iNOS in GPP group was significantly higher than that in GLP group and PUP group. The results indicated that the increased release of NO in GPP might be related to the increased mRNA expression of iNOS. 

We also detected the mRNA expression of related factors such as TNF-α, IL-6 and IL-10 in each group at the concentration of 100 μg/mL. LPS significantly stimulated the mRNA expression of TNF-α and IL-10 (Figure 2b,c). Moreover, the TNF-α mRNA level was significantly stimulated in each experimental group compared with the control group (Figure 2b). Among the three groups, GPP had the strongest effect on stimulating macrophages of TNF-α mRNA expression, although there was no significant difference. Figure 2c showed that three experimental groups also significantly increased the mRNA expression of IL-10, and the PPS group had the strongest effect on stimulating macrophages of IL-10 mRNA expression. There were no significant changes in the expression of IL-6 mRNA (Figure 2d). The results indicated that the immunomodulatory effect of GPP on macrophages included stimulation of some cytokine secretion.

### 3.3. An Overdose of GPP Oral Administration was Safe in Mice

**Figure 3:**
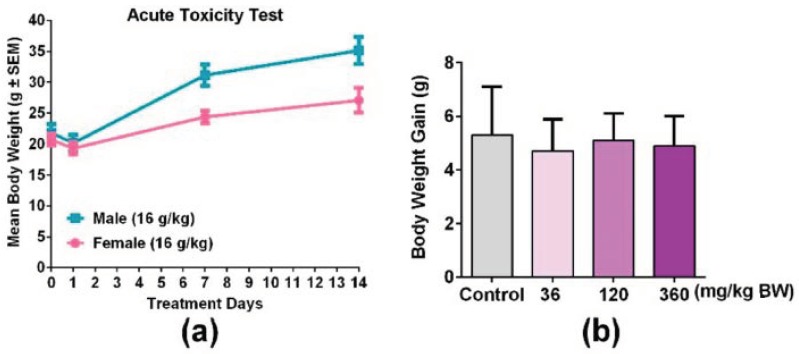
GPP orally administered did not show significant changes in the body weight of mice. (**a**) Male mice and female mice were treated with 16.0 g/kg BW GPP twice per day for 14 days, and the body weight of both genders of mice were recorded on the 0, 1, 7, and 14th day (N = 10). (**b**) Body weight gain of mice after being treated with 36, 120, 360 mg/kg BW of GPP for 30 days in four groups (N = 12). Data were expressed as mean ± SEM.

In order to evaluate the toxicity of GPP, an acute oral toxicity test was detected. The result showed that body weight (Figure 3), respiration, daily behavior changes, death and gross anatomy did not show obvious abnormality, indicating that the half lethal dose (LD50) of GPP was greater than 16.0 g/kg BW. According to the classification standard of acute toxicity of health food, the oral acute toxicity of GPP belongs to the non-toxic grade in mice. In addition, body weight gain was detected during the immune function experiment, and the result showed that the weight of the mice showed a normal growth trend in all groups (Figure 3b).

### 3.4. GPP Did Not Affect the Spleen Index and Thymus Index in Mice

**Figure 4:**
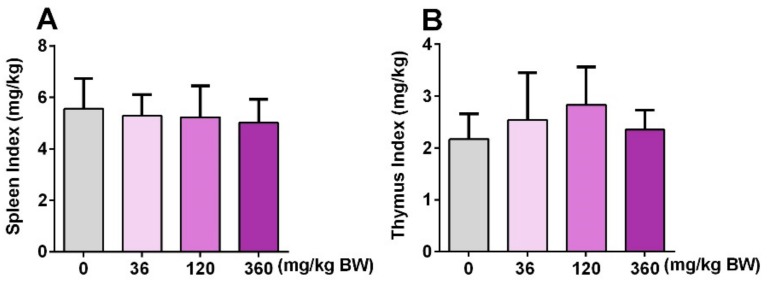
The effect of GPP on immune organ index. The spleen index (**A**) and thymus index (**B**) were detected after treatment with 36, 120, 360 mg/kg BW for 30 days in four groups (N = 12). The spleen or thymus index was calculated by the spleen or thymus weight (mg)/bodyweight (kg). Data were expressed as mean ± SEM (N = 12).

Both spleen and thymus are important immune organs, and the immune organ index is often used as a preliminary indicator to study drugs of immunopharmacology [25]. Therefore, the spleen index and thymus index were detected after mice were treated with different doses of GPP (36, 120, 360 mg/kg BW) for 30 days. The results showed that both the spleen index and thymus in each dose group were not significantly changed compared with the control group (Figure 4), indicating that GPP may not affect the size of the spleen and thymus. 

### 3.5. GPP Increased Peripheral Phagocytes and NK Cell Activity 

**Figure 5:**
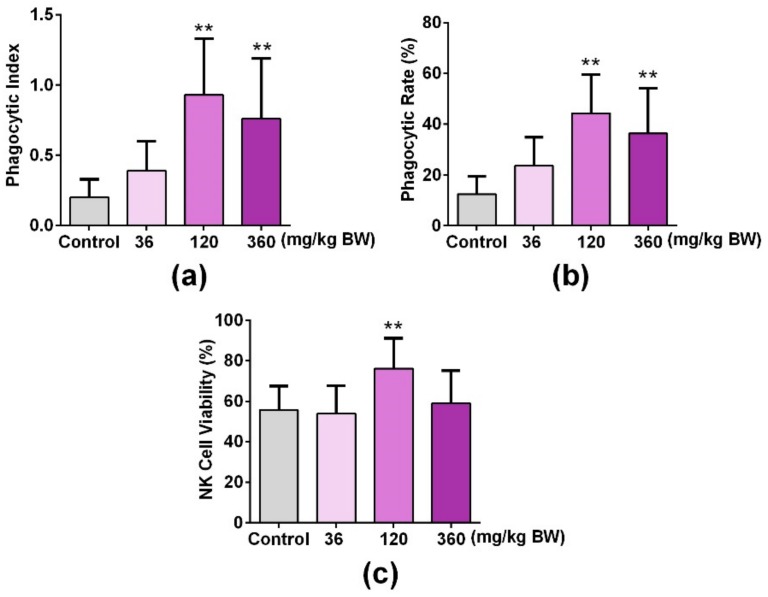
GPP increased peripheral phagocytes and NK cell activity in mice. The uptaking capacity of peripheral phagocytes was detected by mouse peritoneal macrophage phagocyte chicken erythrocytes test, and the (**a**) phagocytic rate and (**b**) phagocytic index represented the phagocytosis of macrophages. (**c**) An LDH releasing assay was used to detect the NK cell activity of the spleen. Data were expressed as mean ± SEM (N = 12). ** *p* < 0.01 vs. control group.

In order to investigate the role of GPP on the innate immune function of mice, the phagocytosis of mouse abdominal macrophages and the NK cell activity of the spleen were detected after mice were administered with GPP at different concentrations (0, 3.6, 120, 360 mg/kg BW) orally for 30 days. As shown in Figure 5a,b, the phagocytic rate and phagocytic index were significantly improved in middle and high dose groups (120, 360 mg/kg) compared with the control group (*p* < 0.01), which suggested that GPP enhanced the phagocytosis of peritoneal macrophages in mice. Moreover, the cytotoxic activity of NK cells against YAC-1 tumor cells was significantly increased in the medium dose (120 mg/kg) group compared with the control group (*p* < 0.01). The above experimental results suggested that GPP was involved in the regulation of innate immunity in mice.

## 4. Discussion

Mushrooms are a food resource which are distributed across the globe and consumed for their nutritional value [26,27]. In addition, mushrooms contain a variety of nutrients, such as carbohydrates, protein, minerals and terpenoids, which are beneficial for human health [28,29,30]. Many bioactive compounds of medicinal mushrooms have been extensively studied including polysaccharides, lectins, lactones, terpenoids and alkaloids [31]. Among them, polysaccharides with multiple activities are the main components of some mushrooms [26]. In recent years, more and more attention has been paid to the immunomodulatory function of mushroom polysaccharides. 

The phagocytosis of macrophages is one of the main mechanisms of body defense against foreign body invasion [32]. NO is one of the main effector molecules produced by macrophages. NO not only enhances the activity of macrophages, but also plays an important regulatory role in innate immunity and adaptive immunity as an important signaling molecule [33]. In this study, we firstly explored whether GPP regulated macrophage function in vitro. The results showed that GPP has the tendency to increase the ability of phagocytic neutral red and release of NO compared with the GLP group (Figure 1). Moreover, GPP significantly enhanced the ability of phagocytosis at the concentrations of 100 and 200 μg/mL (*p* < 0.01, *p* < 0.05), and significantly enhanced the ability of macrophages to release NO at the concentrations of 50 and 100 μg/mL (*p* < 0.05), when compared with the PUP group (Figure 1). The results indicated that GPP enhanced the phagocytic ability and NO production of macrophages compared with GLP or PUP to some extent. According to the above experimental results, the mRNA expression of iNOS, which was closely related to the production of NO, was detected by RT-qPCR. The result (Figure 2a) showed that the expression mRNA of iNOS was significantly increased in each experimental group compared with the control group at the concentration of 100 μg/mL. Moreover, the mRNA expression of iNOS was the highest in the GPP group, indicating that GPP could significantly enhance the mRNA expression of iNOS. 

Macrophages have the characteristic of being heterogeneous, and their phenotypes and functions are regulated by the surrounding microenvironment [34,35]. In response to exogenous stimuli, macrophages polarized into different phenotypes in different stages of inflammatory response [36]. There are mainly two subtypes, namely classical polarization (M1 type) or alternating polarization (M2 type) [36,37]. The equilibrium polarization of macrophages M1/M2 controls the fate of inflammatory or damaged organs. The function of M1 macrophages are involved in the inflammatory response, pathogen clearance, antitumor immunity [38], and enhancing phagocytosis and releasing TNF-α, IL-6 and other pro-inflammatory factors [21,38]. In this study, there was no significant change in IL-6 mRNA expression in each experimental group compared with the control group (Figure 2d). However, the mRNA expression of TNF-α was significantly increased (Figure 2b), and the mRNA expression of TNF-α in the GPP group was higher than that in the GLP group and PUP group, although without statistical significance, indicating that GPP could enhance the mRNA expression of TNF-α. If M1 macrophages are continuously enhanced, they will lead to tissue damage. At this point, M2 macrophages would secrete large amounts of IL-10, arginase-1 (Arg1) and other anti-inflammatory factors to inhibit inflammation, promote tissue repair and reconstruction, angiogenesis, and maintain homeostasis [38]. We found that the mRNA expression of the anti-inflammatory factor IL-10 in each experimental group was significantly enhanced compared with the control group (Figure 2c). The experimental results showed that GPP could regulate the immune function of macrophages from multiple perspectives, but overall enhanced the function of M1 macrophages.

In the animal experiment, the result of the acute oral toxicity test of GPP showed that the LD50 of GPP in mice was greater than 16.0 g/kg body weight (Figure 3a). According to the classification standard of acute toxicity of health food, the oral acute toxicity of GPP belonged to the non-toxic grade. To investigate the effect of GPP on the regulation of immune function, the ICR mice were given GPP (36, 120, 360 mg/kg) at different concentrations by gavage for 30 days, and there was no significant difference in weight gain among the groups (Figure 3b). The spleen index and thymus index could reflect the level of immune function of the body to some extent [39]. The effect of drugs on the thymus index and spleen index can be used as a preliminary indicator to study the mechanism of animal immunopharmacology [25]. In this paper, both the spleen index and thymus index were not significantly changed compared with the control group, suggesting that the GPP did not affect the size of the spleen and thymus (Figure 4b).

The mononuclear macrophage system and NK cells belong to innate immunity. The mononuclear macrophage system is an important part of innate immunity, and is composed of mononuclear cells, macrophages and their precursor cells in bone marrow [40]. Mononuclear cells exist in blood. When mononuclear cells enter tissues through blood vessels, they become macrophages. The NK cell is an important innate immune cell, which has a strong cytolytic function to tumor cells, virus infected cells and other physiological stress cells [41]. NK cell activity is an important indicator of the nonspecific immune system [42]. To investigate their effect on the mouse innate immune function, we tested the phagocytic function of mononuclear macrophages and the activity of NK cells. In the experiment of the peritoneal macrophage phagocytosis of chicken erythrocytes, GPP significantly enhanced the function of mouse peritoneal macrophage phagocytosis of chicken erythrocytes at doses of 120 and 360 mg/kg, suggesting that GPP enhanced the phagocytosis of mouse peritoneal macrophage (Figure 5a,b). In the NK cell viability test, the NK cell activity of mice in the medium-dose (120 mg/kg) group was significantly enhanced compared with that in the control group, suggesting that GPP significantly enhanced the NK cell activity of mice (Figure 5c). The above results showed that GPP could enhance the innate immune function of mice. Our results also showed that GPP could affect the function of macrophages directly or indirectly, which indicated that GPP could affect some signal pathways of macrophages directly or affect the function of macrophages in another way. However, the mechanism of GPP is not yet clear, and needs to be further explored. Some studies have been reported that mushroom polysaccharides could affect immune function by interaction with gut microbiota [4,43,44]; therefore, we will explore the mechanism of GPP by studying the relation between GPP, gut microbiota, and the innate immune system. 

## 5. Conclusions

In summary, the composition of two polysaccharides, GPP, enhanced the phagocytic ability and NO production of macrophages and the expression of iNOS and TNF-α at the RNA level. In addition, GPP significantly enhanced the phagocytic function of macrophages and the activity of NK cells in mice. Based on the above experimental results, we speculate that GPP moderately regulates innate immune function in mice.
